# Descriptive analysis of childbirth healthcare costs in an area with high levels of immigration in Spain

**DOI:** 10.1186/1472-6963-11-77

**Published:** 2011-04-15

**Authors:** Mercè Comas, Laura Català, Maria Sala, Antoni Payà, Assumpció Sala, Elisabeth Del Amo, Xavier Castells, Francesc Cots

**Affiliations:** 1 Epidemiology and Evaluation Department, IMIM-Hospital del Mar, Barcelona, Spain; 2 CIBER Epidemiología y Salud Pública (CIBERESP), Spain; 3 Preventive Medicine and Public Health Educational Unit, Hospital del Mar-Universitat Pompeu Fabra-Agència de Salut Pública de Barcelona (H.MAR-UPF-ASPB), Barcelona, Spain; 4 Universitat Oberta de Catalunya (UOC), Barcelona, Spain; 5 Universitat Autònoma de Barcelona (UAB), Barcelona, Spain; 6 Obstretics and Gynecology Department, IMIM-Hospital del Mar, Barcelona, Spain

## Abstract

**Background:**

The aim of this study was to estimate the cost of childbirth in a teaching hospital in Barcelona, Spain, including the costs of prenatal care, delivery and postnatal care (3 months). Costs were assessed by taking into account maternal origin and delivery type.

**Methods:**

We performed a cross-sectional study of all deliveries in a teaching hospital to mothers living in its catchment area between October 2006 and September 2007. A process cost analysis based on a full cost accounting system was performed. The main information sources were the primary care program for sexual and reproductive health, and hospital care and costs records. Partial and total costs were compared according to maternal origin and delivery type. A regression model was fit to explain the total cost of the childbirth process as a function of maternal age and origin, prenatal care, delivery type, maternal and neonatal severity, and multiple delivery.

**Results:**

The average cost of childbirth was 4,328€, with an average of 18.28 contacts between the mother or the newborn and the healthcare facilities. The delivery itself accounted for more than 75% of the overall cost: maternal admission accounted for 57% and neonatal admission for 20%. Prenatal care represented 18% of the overall cost and 75% of overall acts. The average overall cost was 5,815€ for cesarean sections, 4,064€ for vaginal instrumented deliveries and 3,682€ for vaginal non-instrumented deliveries (p < 0.001). The regression model explained 45.5% of the cost variability. The incremental cost of a delivery through cesarean section was 955€ (an increase of 31.9%) compared with an increase of 193€ (6.4%) for an instrumented vaginal delivery. The incremental cost of admitting the newborn to hospital ranged from 420€ (14.0%) to 1,951€ (65.2%) depending on the newborn's severity. Age, origin and prenatal care were not statistically significant or economically relevant.

**Conclusions:**

Neither immigration nor prenatal care were associated with a substantial difference in costs. The most important predictors of cost were delivery type and neonatal severity. Given the impact of cesarean sections on the overall cost of childbirth, attempts should be made to take into account its higher cost in the decision of performing a cesarean section.

## Background

In some European countries child delivery represents 5% of the entire hospital activity [1], but the healthcare activity and costs generated by childbirth have been insufficiently evaluated.

The cost-effectiveness of prenatal care has been widely described [2-4], although this process among immigrants and its outcomes are controversial. The hypothesis that immigrants make lesser use of prenatal care, which may increase the risk of cesarean section and delivery costs, is widespread [5]. A study in Taiwan found that foreign-born mothers tended to use fewer inpatient services for complicated pregnancies than native-born mothers and were less likely to undergo cesarean section [6]. In Canada, different outcomes for the risk of preterm birth, depending on the length of residence in the country, were found [7]. In Spain, the more disadvantaged neighborhoods have worse pregnancy outcomes, but these inequalities did not exist among immigrant women, and some immigrant groups had better outcomes than Spanish-born women [5].

Catalonia is one of the Autonomous Regions of Spain attracting the greatest number of immigrants [8] most of whom settle in Barcelona, especially in certain areas of the city, Ciutat Vella being the district with the highest percentage of immigrants from non-occidental countries and with the lowest socioeconomic level of all Barcelona districts.

Variation in childbirth cost is principally determined by delivery type, which influences personnel and surgical requirements [9], as well as pre- and postnatal care, especially the postnatal hospital length of stay, both for the newborn and the mother [10,11]. In the last few decades, cesarean section has become more frequent and, at the same time, more controversial. An increase in cesarean sections in low-risk women from 1996 to 2003 was observed according to age, race and ethnicity, although the major risk factor was previous cesarean section [12]. In countries such as the United States, women with a high socioeconomic position have greater access to prenatal care and a higher probability of delivery through cesarean section [13]. The possibility of elective cesarean section, available in some countries, has intensified the debate [14]. Moreover, the debate is also made more intense by the concurrence of increases in medically induced birth and decreased infant and fetal mortality rates [15,16].

The increase in cesarean section rates in non-urgent or not strictly indicated cases unnecessarily increases resource use and poses an additional risk to the safety of the mother and child. Delivery through cesarean section is costlier than non-instrumented vaginal delivery, while instrumented vaginal delivery is more expensive than spontaneous vaginal delivery [17].

Cesarean section rates over 10 or 15%, which are lower than the current average rate in developed countries, do not affect maternal or neonatal morbidity and mortality [18]. High-risk pregnancies are directly associated with gestational age, birthweight and length of hospital stay [19].

For all immigrants living in Spain, the principles of public, free and universal services mainly apply to emergency care services, whereas access to primary and specialized care might present several barriers. These barriers include the need to have a health card, lack of knowledge about how to access these services, and difficulties in making an appointment (language, schedules, waiting list, etc.). To obtain a health card, the only requisite is proof of a place of residence through the registration certificate. Thus, the percentage of unregistered foreign-born residents, although unknown, is believed to be very small.

The aim of this study was to evaluate the overall cost of the childbirth process including the costs of prenatal care, delivery and postnatal care up to 3 months after delivery, taking into account delivery type and maternal origin.

## Methods

### Study population

A cross-sectional study was designed in the area of Hospital del Mar in Barcelona, Spain (a public teaching hospital), and its primary care program for sexual and reproductive health in the Barcelona districts of Ciutat Vella and San Martí, representing a catchment area of 320,000 inhabitants.

All the deliveries in Hospital del Mar from October 2006 to September 2007 were included. We gathered all the public healthcare and economic information on the pregnancy (starting at 9 months before delivery), the delivery itself (including associated maternal and neonatal costs), and data on the mother and child up to 3 months after delivery. As a public service, professionals involved in the childbirth healthcare process provided services to women regardless of their origin or any other characteristic. Foreign-born women were from South America, Asia and the North of Africa, being Morocco, Pakistan and Ecuador the most represented countries.

During the study period there were 1,475 childbirths in Hospital del Mar. A total of 449 deliveries were excluded: 385 (26.1%) because the mother's residence fell outside the catchment area and 64 (4.3%) because data could not be linked among the information systems. Finally, 1,026 childbirths were analyzed.

All the information was treated confidentially. The ethical principles for medical research in human beings defined in the Declaration of Helsinki of 1964 and revised by the World Medical Organization in Edinburgh 2000 and Organic Law 15/1999 of Data Protection in Spain were followed. The study was approved by the ethics comitee of IMIM-Hospital del Mar.

### Cost assessment

The overall cost of a delivery was calculated by taking into account the cost of the activities related to the process including the 9 months of pregnancy prior to delivery, delivery itself and the 3-month postnatal period for both the mother and the newborn. The information sources were the activity records of the Program for Sexual and Reproductive Health, the health care information system and the cost accounting system of Hospital del Mar.

Hospital del Mar uses a hospital cost accounting system based on full-costing allocation. In the present study cost estimation was based on a full-costing accounting system and on the criteria of clinical Activity Based Costing (ABC) methods to obtain maximal sensitivity in the assessment of clinical activity variability. This system ensures that the hospital's total costs are distributed among the patients. Allocation is based on directly assigning the cost of the following services to the patient: laboratory, pharmacy, radiology, nuclear medicine, pathology, prostheses and other diagnostic and therapeutic tests. The information system contains exhaustive data on human resources and their activity: storage, admissions planning, ambulatory and emergency care, operating rooms, diagnostic and complementary tests, and inter-hospital consultations. This information creates and automatically updates the cost for overheads. Fixed cost derived from surgical procedures, hospitalization, and intensive care unit stay, is distributed based on routine criteria: duration of the intervention or the number of stays among the different hospitalization units. The cost predictor variables were age, maternal origin (Spanish-born versus foreign-born), prenatal care (yes or no), delivery type [according to the ICD-9: vaginal non-instrumented (73.4 and 73.59); instrumented (72.XX, 73.1, 73.2, 73.3 and from 73.80 to 73.99) and cesarean section (74.XX)], multiple pregnancy and maternal and neonatal severity (measured by the APR-DRG [20]) severity index. The APR-DRG severity index was based on the comorbidites included in the secondary diagnostics used to calculate the APR-DRGs. For newborns, it was available for admitted newborns only. All newborns are registered after birth, but only those newborns needing tests or clinical assistance are admitted to the hospital.

### Statistical analysis

The unit of analysis was maternal admission. A Chi-square test was used to compare categorical variables. The Kruskal-Wallis and Mann-Whitney U tests were used to compare continuous variables. Partial costs of the process and the number of acts were analyzed overall and by maternal origin and delivery type. Overall cost was analyzed by maternal age, maternal origin, prenatal care, delivery type, postnatal care, maternal and neonatal severity and multiple pregnancy. Overall cost was normalized through the log and the inverse transformations, lending the inverse transformation a closer approximation to normality. Multivariate analyses of hospital cost were performed using generalized linear models with inverse-transformed costs. All the variables significantly related to the outcome of interest (p < 0.20) were included in the multivariate model. Final multivariate models were adjusted by maternal age, maternal origin, pregnancy follow-up, delivery type, maternal and neonatal severity score (according to APR-DRG severity of illness subclass [20]), and multiple delivery. After fitting the model, predictions of the overall inverse-transformed cost were calculated for each category of the predictor variables, while the remaining variables were kept at the reference level (15 for age, Spanish-born woman for origin, yes for prenatal care, vaginal non-instrumented for delivery type, mild for maternal severity, non-admitted to hospital for neonatal severity and single for multiple birth). To interpret the results of the model, these predictions were transformed back to euros. The value of the constant of the model corresponded to the cost of a vaginal non-instrumented delivery of a 15-year-old Spanish-born woman with prenatal care and low severity, who delivered a singleton not admitted to hospital. By using the estimated overall cost, the incremental cost and its weight (%) for each category of the predictor variables was calculated.

The level of statistical significance was set at 0.05 and all tests were two-tailed. Analyses were conducted using SPSS, version 13.0 (SPSS, Chicago, IL, USA, 2003).

## Results

Table 1 describes the 1,026 deliveries, overall and according to maternal origin. Compliance with the prenatal care program at primary care was significantly more frequent in foreign-born mothers (80.1% vs 71.9% of Spanish-born mothers, p = 0.002). More than 27% of deliveries were through cesarean section in both groups. Although differences were not statistically significant (p = 0.722), foreign-born mothers had a 2.2% higher proportion of vaginal non-instrumented deliveries than Spanish-born mothers, while Spanish-born mothers had a 1.5% higher proportion of vaginal instrumented deliveries. Spanish-born mothers had a worse severity profile (p = 0.002). Differences in neonatal severity were not statistically significant (p = 0.332), but the percentage of newborns admitted to the hospital was 4.9% higher among foreign-born mothers.

**Table 1:** Descriptives of the whole sample of women, stratified by origin (1,026 women and 1,053 newborns)

	Overall (N = 1,026)		Spanish-born mothers (N = 462)		Foreign-born mothers (N = 564)		
		
	n	%	n	%	n	%	p*
Age [mean (SD)]	30.4 (6.0)		30.25 (6.2)		30.54 (5.9)		0.444
							
Prenatal care	784	76.4	332	71.9	452	80.1	0.002
							
Delivery type							
Vaginal non-instrumented	575	56.0	253	54.8	322	57.0	
Vaginal instrumented	171	16.7	81	17.5	90	16,0	
Cesarean section	280	27.3	128	27.7	152	27.0	0.722
							
Maternal severity							
Mild	519	50.6	208	45,0	311	55.1	
Moderate	480	46.8	237	51.3	243	43.1	
Severe	27	2.6	17	3.7	10	1.8	0.002
							
Neonatal severity							
Non-admitted	323	31.5	158	34.2	165	29.3	
Mild	609	59.3	266	57.6	343	60.8	
Moderate	81	7.9	32	6.9	49	8.7	
Severe	13	1.3	6	1.3	7	1.2	0.332
							
Multiple birth	26	2.5	15	3.2	11	1.9	0.232

SD: Standard Deviation. * Exact Chi-square test.

The average cost of pre- and postnatal care and delivery was 4,328€. No statistically significant difference was observed according to maternal origin (Table 2). There were an average of 18.28 contacts between the mother and neonate with the healthcare facilities (Table 2). The highest proportion of contacts corresponded to prenatal visits to primary care (31.7%) and hospital contacts, including tests (19.7%), visits (13.2%) and emergencies (10.3%). Maternal hospital admission for the delivery itself (only 1 act per woman, 5.5% of the overall number of acts) accounted for the highest proportion of the overall cost (56.7%), followed by neonatal admission (an average of 1.03 acts, 5.6% of acts), accounting for 19.9% of the overall cost.

**Table 2:** Healthcare acts and costs per delivery, overall and stratified by process, for the whole sample and by maternal origin

	Overall (N = 1,026)				Spanish-born mothers (N = 462)				Foreign-born mothers (N = 564)				p of acts	p of cost
	
Process	Acts	%	Costs (SD)	%	Acts	%	Costs (SD)	%	Acts	%	Costs (SD)	%		
														
Pregnancy														
Primary care visits	5.79 (4.4)	31,7	137.92 (99.3)	3,2	5.80 (4.7)	30,6	136.87 (106.9)	3,2	5.79 (4.1)	32,6	138.79 (92.6)	3,2	0,556	0,566
Emergencies	1.88 (2.2)	10,3	164.65 (200.1)	3,8	2.18 (2.6)	11,5	191.49 (228.7)	4,4	1.63 (1.9)	9,2	142.66 (170.3)	3,3	0,001	0,002
Hospital contacts	2.41 (3.3)	13,2	276.10 (491.8)	6,4	2.50 (3.1)	13,2	241.12 (431.0)	5,6	2.33 (3.4)	13,1	304.75 (535.2)	7,0	0,042	0,806
Tests	3.59 (2.6)	19,7	212.17 (242.0)	4,9	3.81 (2.7)	20,1	237.41 (273.9)	5,5	3.41 (2.5)	19,2	191.5 (210.2)	4,4	0,009	0,007
														
Delivery														
Maternal admission	1	5,5	2,453.48 (819.4)	56,7	1	5,3	2,474.42 (823.7)	57,5	1	5,6	2,436.32 (816.1)	56,1		0,050
Neonatal admission	1.03 (0.2)	5,6	859.48 (1,575.4)	19,9	1.03 (0.2)	5,5	825.56 (1,321.1)	19,2	1.02 (0.1)	5,7	887.26 (1,757.2)	20,4	0,187	0,325
														
Three-month postnatal care														
Primary care visits	1.44 (1.5)	7,9	30.69 (30.9)	0,7	1.50 (1.6)	7,9	32.14 (33.2)	0,7	1.39 (1.4)	7,8	29.51 (28.9)	0,7	0,617	0,495
Hospital contacts														
Newborn	0.80 (1.3)	4,4	139.35 (526.7)	3,2	0.75 (1.2)	4,0	126.86 (456.8)	2,9	0.84 (1.3)	4,8	149.58 (577.9)	3,4	0,231	0,250
Mother	0.32 (1.0)	1,8	52.37 (566.1)	1,2	0.34 (0.9)	1,8	39.90 (173.4)	0,9	0.31 (1.1)	1,7	62.59 (747.4)	1,4	0,211	0,229
Tests	0.02 (0.4)	0,1	1.60 (35.7)	0,0	0.01 (0.2)	0,1	0.89 (15.3)	0,0	0.03 (0.6)	0,2	2.19 (46.2)	0,1	0,908	0,905
														
Total per delivery	18.28 (8.1)	100	4,327.82 (2,377.3)	100	18.93 (8.3)	100	4,306.67 (1,974.1)	100	17.75 (7.9)	100	4,345.15 (2,664.1)	100	0,004	0,079
Total for all deliveries	18.756		4.440.344,12		8.745		1.989.679,43		10.011		2.450.664,68			

Acts and costs are expressed as means (standard deviation). Percentages are calculated over the total amount of activity or costs. P-values for Mann-Whiteny U tests.

The profile of pregnancy follow-up differed according to maternal origin (Table 2). Spanish-born mothers had a statistically significantly higher average number of emergencies (p = 0.001), tests (p = 0.009) and hospital contacts (p = 0.042). These differences were also found in the costs for emergencies (p = 0.002) and tests (p = 0.007). The difference in the cost of hospital contacts was in the opposite direction, the average cost being 63.63€ higher in foreign-born mothers, but this difference was not statistically significant. No other statistically significant differences were found according to maternal origin, except for the overall number of acts per delivery, Spanish-born mothers having an average of 1.18 more acts per delivery (p = 0.004).

The average overall cost of a delivery differed according to delivery type (Table 3) and was 5,815€ for cesarean section, 4,064€ for vaginal instrumented delivery and 3,682€ for vaginal non-instrumented delivery (p < 0.001 for the 3-group and all 2-by-2 comparisons). The average number of acts also differed among groups (p < 0.001), due to the difference of vaginal non-instrumented delivery compared with vaginal instrumented delivery (p = 0.005, a difference of 2.16 fewer acts) and compared with cesarean section (p < 0.001, a difference of 3.64 fewer acts).

**Table 3:** Healthcare acts and costs per delivery, overall and stratified by process and delivery type

	Vaginal non-instrumented (N = 575)				Vaginal instrumented (N = 171)				Cesarean section (N = 280)				p of acts	p of cost
	
Process	Acts	%	Costs (SD)	%	Acts	%	Costs (SD)	%	Acts	%	Costs (SD)	%		
														
Pregnancy														
Primary care visits	5.55 (4.3)	32,8	132.39 (97.9)	3,6	6.02 (4.4)	31,5	143.46 (100.0)	3,5	6.14 (4.5)	29,8	145.92 (101.3)	2,5	0,089	0,091
Emergencies	1.79 (2.3)	10,6	155.49 (197.1)	4,2	2.08 (2.4)	10,9	187.70 (217.7)	4,6	1.94 (2.1)	9,4	169.38 (194.4)	2,9	0,100	0,044
Hospital contacts	2.08 (2.7)	12,3	247.24 (470.1)	6,7	2.42 (3.1)	12,7	333.13 (638.9)	8,2	3.06 (4.3)	14,9	300.53 (425.3)	5,2	0,017	0,012
Tests	3.24 (2.5)	19,1	187.99 (226.5)	5,1	3.58 (2.6)	18,8	206.50 (228.0)	5,1	4.31 (2.8)	21,0	265.28 (271.7)	4,6	<0.001	<0.001
														
Delivery														
Maternal admission	1	5,9	2,104.56 (311.7)	57,2	1	5,2	2,360.64 (385.8)	58,1	1	4,9	3,226.70 (1,148.7)	55,5		<0.001
Neonatal admission	1.02 (0.1)	6,0	683.36 (1,370.5)	18,6	1.01 (0.1)	5,3	637.21 (433.2)	15,7	1.05 (0.2)	5,1	1,356.89 (2,190.1)	23,3	0,072	<0.001
														
Three-month postnatal care														
Primary care visits	1.34 (1.5)	7,9	28.72 (31.1)	0,8	1.60 (1.5)	8,4	34.29 (31.0)	0,8	1.54 (1.4)	7,5	32.54 (30.5)	0,6	0,014	0,018
Hospital contacts														
Newborn	0.76 (1.3)	4,5	127.37 (461.0)	3,5	0.87 (1.2)	4,6	104.11 (328.4)	2,6	0.86 (1.3)	4,2	185.47 (716.2)	3,2	0,026	0,012
Mother	0.13 (0.4)	0,8	14.54 (101.7)	0,4	0.49 (1.3)	2,6	55.44 (219.2)	1,4	0.62 (1.4)	3,0	128.18 (1,057.4)	2,2	<0.001	<0.001
Tests	0.01 (0.1)	0,1	0.31 (3.7)	0,0	0.02 (0.3)	0,1	1.89 (24.7)	0,0	0.05 (0.8)	0,2	4.08 (65.4)	0,1	0,986	0,986
														
Total per delivery	16.93 (7.6)	100	3,681.98 (1,747.9)	100	19.09 (8.0)	100	4064.38 (1,222.1)	100	20.57 (8.7)	100	5,814.98 (3,231.9)	100	<0.001	<0.001
Total for all deliveries	9.733		2.117.138,99		3.264		695.009,72		5.759		1.628.195,40			

Acts and costs are expressed as means (standard deviation). Percentages are calculated over the total amount of activity or costs. P-values for Kruskal-Wallis tests.

The most important difference in costs was related to the cost of maternal admission for the delivery itself, cesarean section being 866.06€ costlier than vaginal instrumented delivery (p < 0.001) and vaginal instrumented delivery being 256.08€ costlier than vaginal non-instrumented delivery (p < 0.001). The cost of neonatal admission also differed significantly according to delivery type, the average being 1,356.89€ for cesarean section (23.3% of the overall cost), 683.36€ for vaginal non-instrumented delivery and 637.21€ for vaginal instrumented delivery (all 2-by-2 comparisons were significant at the 0.05 level).

The type of delivery was associated with significant differences in the number of acts and the cost of postnatal care for the 3 months after delivery in all concepts except for tests, which was the least frequent concept. The 2-by-2 differences were significant at the 0.05 level for all concepts between cesarean sections and vaginal non-instrumented delivery, and between vaginal instrumented and vaginal non-instrumented deliveries. When cesarean sections were compared with vaginal instrumented deliveries, only the mother's hospital contacts showed significant differences in the number of acts and costs (Table 3).

No significant differences among delivery types were found for prenatal care at primary care, the concept with the highest weight in terms of the number of acts (around 31%). Although the average number of emergency visits was similar (around two), their cost for vaginal instrumented deliveries was 32.21€ higher than that for non-instrumented deliveries (p = 0.025). Women who underwent cesarean section contacted the hospital once more than women with a vaginal non-instrumented delivery (p = 0.007). However, these hospital contacts were costlier for vaginal instrumented deliveries, with differences of 85.89€ compared with vaginal non-instrumented deliveries (p = 0.031) and 53.29€ compared with cesarean sections (p = 0.011). Cesarean sections were associated with a significantly higher number and cost of tests, with differences of 0.73 and 1.07 tests (p = 0.005 and p < 0.001) and 58.78€ and 77.29€ (p = 0.007 and p < 0.001) with vaginal instrumented and vaginal non-instrumented deliveries, respectively.

Table 4 shows the estimated average total cost of deliveries according to the categories of the covariables. The multivariate model explained 45.5% of the variability of the cost. The estimated overall cost of a vaginal non-instrumented delivery in a 15-year old Spanish-born woman with prenatal care and mild severity, who delivered a single newborn who was not admitted to hospital was 2,992€ with a 95% confidence interval from 2,881 to 3,112. The incremental cost for a delivery through cesarean section was 955€ (an increase of 31.9%) versus an increase of 193€ (6.4%) for an instrumented vaginal delivery. The incremental cost of admitting the newborn to the hospital ranged from 420€ (14.0%) to 1,951€ (65.2%), depending on the newborn's severity. The remaining covariables were not statistically significant or economically relevant (Table 4).

**Table 4:** Multiple linear regression on the inverse of the overall cost (N = 1,026).

	**Incremental cost (**€**)**			
		
	**Estimate (**€**)**	**95% Confidence Interval (**€**)**	Variation (%)**	p
				
Reference case*	2,992.12	[2,880.77; 3,112.42]		<0.001
				
Age (per 1 year increase over 15)	5.09	[-0.40; 11.49]	0.17	0.071
				
Origin				
Spanish-born mother	Reference			
Foreign-born mother	-72.02	[-123.85; -8.61]	-2.41	0,028
				
Prenatal care				
Yes	Reference			
No	-189.70	[-238.16; -129.62]	-6.34	<0.001
				
Delivery type				
Vaginal non-instrumented	Reference			
Vaginal instrumented	192.80	[81.04; 331.96]	6.44	<0.001
Cesarean section	954.47	[753.80; 1,204.79]	31.90	<0.001
				
Maternal severity				
Mild	Reference			
Moderate	241.28	[148.43; 354.69]	8.06	<0.001
Severe	597.24	[293.34; 1,017.17]	19.96	<0.001
				
Neonatal severity				
Non-admitted	Reference			
Mild	419.50	[302.95; 562.24]	14.02	<0.001
Moderate	659.40	[435.67; 949.49]	22.04	<0.001
Severe	1,951.41	[1,146.41; 3,287.30]	65.22	<0.001
				
Multiple birth				
Single	Reference			
Multiple	701.34	[375.43; 1,153.94]	23.44	<0.001

The table shows the incremental cost attributable to the variables included in the model with an explained variance of 45.5%.

*: Vaginal non-instrumented delivery of a 15-year old Spanish-born woman with prenatal and mild severity, delivering a singleton not admitted to hospital. **: Variation (%) with respect to the reference case.

## Discussion

The average cost of the childbirth process was 4,328€, with an average of 18.28 contacts of the mother or the newborn with the healthcare facilities. The delivery itself accounted for more than 75% of the overall cost: maternal admission for 57% and neonatal admission for 20%. Prenatal care accounted for 18% of the overall cost and represented 75% of overall acts.

As reported by other studies [5,21,22], no differences were found in overall cost or health services utilization between Spanish-born and foreign-born mothers. These studies reported that utilization rate and adjusted cost were lower in the immigrant population than in the Spanish-born population. The reasons explaining these results in the Spanish context are the healthy immigrant effect and the high labor activity rate. However, foreign-born mothers had a higher frequency of primary care prenatal care (80%) which in this context suggests that it may not be the case that foreign-born mothers find barriers to accessing primary care gynecology services.

Spanish-born mothers had a worse severity profile and a higher average number of emergencies, hospital contacts and tests per woman during pregnancy. The cost per woman related to emergencies and tests was significantly higher for Spanish-born women, although the cost for hospital contacts was higher for foreign-born women. Although 5% more newborns from foreign-born mothers were admitted to hospital, no differences in costs or the number of acts were found for delivery or the 3-month postnatal period. In agreement with our results, a study in Taiwan likewise finds no evidence for the hypothesis that immigrant mothers have worse neonatal outcomes [23].

Given that delivery accounted for the highest proportion of cost, the main predictor of the overall cost of the process was delivery type. The average overall costs were 5,815€ for cesarean sections, 4,064€ for vaginal instrumented delivery and 3,682€ for vaginal non-instrumented delivery. Vaginal non-instrumented deliveries had a lower overall number of acts than the other delivery types and a lower burden of postnatal care. In fact, the average hospital stay in our sample was similar to that in 2008 at the public network of Catalonia, which was 2.71 days for vaginal deliveries and 4.57 days for cesarean sections. The costs of newborns admitted to hospital after a cesarean section doubled the cost of the newborn's admission after a vaginal delivery. This finding may be due to the longer hospital stay of the newborn (and mother) after a cesarean section, but also to the higher frequency of multiple births under cesarean section (4.3% vs. 2.1% for vaginal non-instrumented deliveries). Indeed, a study in women with prior cesarean section found higher rates of respiratory morbidity and neonatal intensive care unit admission, and longer length of hospital stay for cesarean sections than for vaginal births [24]. Cesarean section also affected maternal hospitalization: a study on births in Massachusetts found a higher adjusted likelihood of rehospitalization within 30 days postpartum among women undergoing planned cesarean [25].

The differences between the total cost of the process according to delivery type was clearly significant, as seen in other studies (cesarean section was more expensive than instrumented delivery, and the latter was more expensive than non-instrumented delivery), [3,8,10,17,24].

By using a multivariate regression model, the incremental cost of the different characteristics of the births was calculated. This model stressed the importance of the overall cost of the delivery itself. The type of delivery, maternal severity, neonatal admission and severity, and multiple births had a substantial impact on the individual cost of the delivery. In contrast, prenatal care, maternal origin and age had a slight impact on overall cost.

Nevertheless, for both Spanish and foreign-born mothers the rate of cesarean sections was around 27%. The World Health Organization considers that the optimum rate is unknown, despite the growing body of research that shows a negative effect of high rates of cesarean sections [26]. Cesarean rate may vary according to organizational issues, clinical criteria or mother's preferences however, the economic impact of performing a cesarean section should also be taken into account in the decision-making process of indicating or not a cesarean section.

The assessment of the costs presented in this study was based on the hospital's own information system, which limits the extrapolation of results. The sample analyzed does not allow conclusions to be extrapolated but do describe what is relevant and what is not. However, the quality of costs from a hospital cost accounting system is higher than could have been expected from a general but approximate sectorial study.

Another limitation of this study was the lack of information on the features and costs of women who choose to undergo prenatal care in private health services, this could affect mainly the analysis of maternal origin, as Spanish-born women may have greater access to private healthcare. Nevertheless, gynecologists in our hospital perceive that women who undergo pregnancy care in the private sector do not give birth in a public hospital.

This study has demonstrated that health care information systems in the public sector can be used to describe a process as a whole, beyond partial episodes. Evaluating the cost of maternal hospital stay is necessary but not sufficient to assess the cost of the overall process of delivery. Knowing the cost of the total process (including pregnancy, delivery and postnatal care) and the importance of each episode may help in decision-making on the organization of public healthcare facilities and their relation with populational features.

## Conclusions

Healthcare cost information systems are useful to achieve a complete and detailed cost of the childbirth process. Our results suggest that neither immigration nor pregnancy care are associated with a substantial difference in the overall cost of childbirth. The most important cost predictors are type of delivery, especially cesarean section, and neonatal severity. The rate of cesarean sections was high and similar between Spanish and foreign-born women. Given its importance, the economic impact of cesarean section on the overall cost of childbirth should be factored when considering this mode of delivery.

## Competing interests

The authors declare that they have no competing interests.

## Authors' contributions

FC, MS, AP, AS, EA, XC conceived the idea for the study. FC and MC obtained the data. MC completed the analysis. LC and MC wrote the manuscript which was reviewed by all authors. All authors had full access to all data in the study and can take responsibility for the integrity of the data and the accuracy of the data analysis. All the authors read and approved the final manuscript.

## Pre-publication history

The pre-publication history for this paper can be accessed here:

http://www.biomedcentral.com/1472-6963/11/77/prepub
